# Novel dalbavancin-PLLA implant coating prevents hematogenous *Staphylococcus aureus* infection in a minimally invasive mouse tail vein model

**DOI:** 10.3389/fbioe.2022.1021827

**Published:** 2022-11-17

**Authors:** Marlen Kloss, Caroline Moerke, Franziska Woitschach, Katharina Wulf, Sabine Illner, Steffen Schulz, Viktoria I. Pauker, Katharina Riedel, Niels Grabow, Hüseyin Ince, Emil C. Reisinger, Martina Sombetzki

**Affiliations:** ^1^ Division of Tropical Medicine and Infectious Diseases, Center of Internal Medicine II, University Medical Center Rostock, Rostock, Germany; ^2^ Research Institute for Farm Animal Biology, Dummerstorf, Rostock, Germany; ^3^ Institute for Biomedical Engineering, University Medical Center Rostock, Rostock, Germany; ^4^ EUFH Campus Rostock, University of Applied Science, Rostock, Germany; ^5^ Institute of Microbiology, University of Greifswald, Greifswald, Germany; ^6^ Rectorate, University of Greifswald, Greifswald, Germany; ^7^ Division of Cardiology, Center of Internal Medicine II, University Medical Center Rostock, Rostock, Germany

**Keywords:** hematogenous implant-related infections, cardiovascular implants, *in vivo* biofilm model, antibiotic coating, dalbavancin, rifampicin/minocycline

## Abstract

Infective/bacterial endocarditis is a rare but life-threatening disease with a hospital mortality rate of 22.7% and a 1-year mortality rate of 40%. Therefore, continued research efforts to develop efficient anti-infective implant materials are of the utmost importance. Equally important is the development of test systems that allow the performance of new materials to be comprehensively evaluated. In this study, a novel antibacterial coating based on dalbavancin was tested in comparison to rifampicin/minocycline, and the suitability of a recently developed mouse tail vein model for testing the implant coatings was validated. Small polymeric stent grafts coated with a poly-L-lactic acid (PLLA) layer and incorporated antibiotics were colonized with *Staphylococcus* (*S.*) *aureus* before implantation into the tail vein of mice. The main assessment criteria were the hematogenous spread of the bacteria and the local tissue reaction to the contaminated implant. For this purpose, colony-forming units (CFU) in the blood, spleen and kidneys were determined. Tail cross sections were prepared for histological analysis, and plasma cytokine levels and expression values of inflammation-associated genes were examined. Both antibiotic coatings performed excellently, preventing the onset of infection. The present study expands the range of available methods for testing the anti-infectivity of cardiovascular implants, and the spectrum of agents for effective surface coating.

## 1 Introduction

The development of cardiovascular implants such as intravascular or cardiac devices is considered a historical milestone in the therapy of cardiovascular diseases. However, even with growing advances in material quality and technology, these devices are still associated with life-threatening risks such as implant-related infective endocarditis (Sohail et al., 2008; Le Dolley et al., 2010, 2010; Roig et al., 2012), in-stent restenosis (Alfonso et al., 2014; Buccheri et al., 2016) and thrombosis (Dukkipati et al., 2018). Therefore, it is of the utmost importance to advance research in the field of implant development to minimize the risk of complications.

Conventional, artificial implants have been reported to be more susceptible to bacterial adhesion than natural tissues (Montanaro et al., 2011; Song et al., 2013). This is related to an inevitable foreign body reaction (FBR) induced by the surgical procedure and the implants themselves. Regions of FBR appear in conjunction with a local immunodepression known as *locus minoris resistentiae* (Campoccia et al., 2006; Arciola et al., 2018). It has been shown that under the influence of an FBR, a bacterial load lower by approximately 10^6^ colony-forming units (CFU) than the bacterial load necessary to produce infection in natural tissue is sufficient to manifest an infection (Zimmerli et al., 1982). In early implant infections, which usually occur within three months of surgery, the bacteria originate from perioperative contamination of the implant or surgical site. In late infections, the bacteria are more likely to originate from the bloodstream, for example, when bacteria circulate temporarily in the blood as a result of infections at other sites of the body (LaPorte et al., 1999; Trampuz and Widmer, 2006). Despite extensive efforts to ensure an aseptic environment and aseptic techniques, pathogenic microorganisms can still be detected in approximately 90% of implant sites (Hetrick and Schoenfisch, 2006; Oliveira et al., 2018; Scialla et al., 2021). Beyond that, the growth in the number of long-term intravenous therapies and invasive procedures being performed has led to an increase in staphylococcal bacteremia (Pittet and Wenzel, 1995; Lyytikäinen et al., 2005; Ammerlaan et al., 2013), which is one of the most common precursors of infective endocarditis (IE) (Cahill and Prendergast, 2016). Endocarditis of prosthetic valves accounts for up to 35% of all cases of IE (Blue et al., 2012). In this context, Gram-positive staphylococci, streptococci, and enterococci account for 80–90% of cases, with *Staphylococcus* (*S.*) *aureus* the most commonly detected pathogen with a prevalence of 30% (Murdoch et al., 2009; Selton-Suty et al., 2012). Aggregation in a biofilm is an important virulence factor for these microorganisms. Due to the complex composition of the polysaccharide- and protein-containing matrix in which they live, the bacteria are protected from antibiotics, host immune defenses, and the external physical or chemical environment (Anderl et al., 2000; Stewart and William Costerton, 2001; Wang et al., 2021). This architectural and molecular defense mechanism makes successful treatment difficult, so that removal of the implant is the only remaining therapeutic option in most cases.

The effective functionalization of cardiovascular and other implants is a steadily growing area of research. Starting from bare metal or polymer implants, the field has expanded to include the incorporation of anti-microbial agents (Goëau-Brissonnière et al., 2011; Lew and Moore, 2011; Reitzel et al., 2020), other active biological or physical components (Hårdhammar et al., 1996; Shinozaki et al., 2005) and structural surface modification (Wang et al., 2020). A number of effective approaches involve the use of antibiotics, one being the combined coating of catheters with rifampicin/minocycline, which is known to reduce bloodstream infections (Reitzel et al., 2020) and has been shown to be highly effective against staphylococcal colonization of catheters (Raad et al., 1995; Raad et al., 1996). The use of this antibiotic combination in the absorbable antibacterial envelope TYRX^™^ (Medtronic, Inc. Minneapolis, MN, United States) reduces implantable electronic heart device (CIED) infections too (Boriani et al., 2021). However, widespread, sometimes systemic, therapeutic and prophylactic use of antibiotics has led to the emergence of multidrug-resistant germs which pose a major research challenge and require the development of effective means to combat them (Berberich and Sanz-Ruiz, 2019). Dalbavancin, a semisynthetic lipoglycopeptide antibiotic, exhibits potent *in vitro* bactericidal activity against Gram-positive pathogens including methillicin-resistent *S. aureus* (MRSA), methillicin-resistent *S. epidermidis* (MRSE), and vancomycin-resistent enterococci (VRE) (Streit et al., 2004; Knafl et al., 2017). Furthermore, clinical data indicate that dalbavancin is a safe, effective, and well-tolerated therapy for skin and soft tissue infections (Seltzer et al., 2003; Barberán et al., 2021; Monteagudo-Martínez et al., 2022).

In parallel with the optimization of implant materials, the development of test model systems that physiologically replicate the environment of implants is becoming increasingly important. State-of-the-art research on materials for various other applications, including cardiovascular implants, generally involves subcutaneous animal models (Nakamoto et al., 1995; Roehrborn et al., 1995; Nejadnik et al., 2008). This is problematic because the circulatory system constitutes an environment with numerous physiological variables that are seldom represented in animal models. In addition, there is a drive to use large animal models as they are closer to human physiology. In the cardiovascular context, suitable bovine, porcine or ovine models exist to evaluate appropriate strategies for the treatment of cardiovascular diseases (Monreal et al., 2014). However, these are costly and should therefore only be used if complex prototypes are to be tested. Small animal models are easier to implement and are very useful to get a first impression regarding the host’s response to different implant features (material, shape, active ingredients). An example is the study by Mueller et al. (2012) in which the degradation process of iron foils was investigated in a tail vein model in mice. The unique feature of this study was the minimally invasive insertion of the material without surgical intervention and the presence of physiological blood contact.

We expanded this study and developed an *in vivo* contamination model to test the anti-microbial potential of implant materials (Moerke et al., 2021). In association with the validation of the *in vivo* model, the aim of this present study was to compare a novel antibiotic coating based on dalbavancin as a long shelf-life single agent with a well described local drug depot of rifampicin and minocycline. We hypothesized that the integration of dalbavancin into a poly-L-lactic acid (PLLA) coating for polymeric stent grafts would prevent the hematogenous spread of bacteria originating from implant infection.

## 2 Materials and methods

### 2.1 Ethical statement

Animal experiments were carried out in strict accordance with the regulations of the German Society for Laboratory Animal Science and with the European health guidelines issued by the Federation of Laboratory Animal Science Associations. The protocol was approved by the local animal care and use committee (7221.3-1-069/19). All efforts were made to minimize animal suffering.

### 2.2 Fabrication and characterization of material samples

For *in vitro* studies, glass slides (approximately 25.5 mm × 75.5 mm) were spray coated either with poly-L-lactic acid (PLLA; Resomer L210, Evonik Industries AG, Germany) only, PLLA + rifampicin/minocycline or PLLA + dalbavancin. Rifampicin, minocycline (Merck KGaA, Germany) and dalbavancin (Oskar Tropitzsch, Germany) were dissolved in chloroform, mixed with PLLA and sprayed as a mixture onto glass slides. The coating solutions contained 20% combined rifampicin and minocycline (1,200 mg/L, each) or 10% dalbavancin (400 mg/L) based on PLLA. The airbrush procedure was performed using an 0.25 wt% PLLA spray solution under clean room conditions and according to specified process parameters. After processing, the samples were dried in a vacuum drying chamber for 1 week at 37°C. For *in vivo* studies, stent grafts in the form of small tubes were extruded from a thermoplastic silicone polycarbonate elastomer (TSPCU; ChronoSil 80A, AdvanSource Biomaterials, United States) with a micro compounder (HAAKE MiniLab II, Thermo Fisher Scientific, Germany). The same parameters were used for coating the tubes as for the glass slides. Tube diameters were measured before and after the coating process using a biaxial laser scanner (ODAC 32 XY, Zumbach Electronic AG, Switzerland). For quality assurance, the surfaces of the PLLA-coated implants were examined in a QUANTA FEG 250 scanning electron microscope (SEM, Thermo Fisher Scientific, FEI Company, Germany) at different magnifications.

### 2.3 Origin and culture conditions of *Staphylococcus aureus* FR20

*Staphylococcus* (*S.*) *aureus* FR20 was kindly provided by the Department of Microbial Physiology and Molecular Biology at the University of Greifswald, Germany and was originally isolated by the Department of Immunology, University Medicine Greifswald, Germany. The strain originates from a patient with infective endocarditis and following virulence factors were determined: spa-type t008, CC8; superantigen-genes sed, sej, ser; agr-type agr1; hemolysin hla, hld, hlgv. It was cultivated in Luria broth (LB; Sigma-Aldrich, United States) at 37°C with shaking at 150 rpm. For all experiments, the inoculum was prepared by diluting an overnight bacterial broth culture 1:20 in LB medium. For further processing, the concentration of the pre-culture was adjusted to an OD_600_ (optical density measured at a wavelength of 600 nm) of 0.1 or 0.5 after 2–3 h. OD_600_ was measured in a 96-well plate using a micro-plate reader (FLUOstar Omega, BMG LABTECH GmbH, Germany).

### 2.4 *In vitro* anti-microbial activity of rifampicin/minocycline and dalbavancin on *Staphylococcus aureus* FR20 biofilm formation

*In vitro* biofilm analyses were carried out using *S. aureus* FR20 transformed with a green fluorescent protein (GFP)-producing plasmid, pCtuf-gfp, originated by Biswas, 2006 (Biswas, 2006). The *in vitro* antibiotic activity of rifampicin/minocycline and dalbavancin against *S. aureus* FR20 biofilm formation was analyzed in a continuous flow system adapted from Graf et al., 2019 (Graf et al., 2019) using the coated glass slides. In brief, the flow system consisted of a medium bottle, a multichannel pump (Watson-Marlow 205S peristaltic pump; Watson-Marlow GmbH, Rommerskirchen, Germany), a drop trap, flow chambers (Sticky slide I0.8 Luer, 80198 Ibidi) and a waste bottle. The coated glass slides were attached to the bottomless sticky flow chambers and connected to the flow system. Flow chambers were filled with LB medium overnight to equilibrate. Next day, each flow chamber was inoculated with 300 µl of a bacterial culture (OD_600_ = 0.01) using a small syringe. To allow bacterial attachment, the flow chambers were left without flow for 1 h. The flow system was filled with LB medium and subsequent biofilm cultivation was performed with a flow rate of 3 ml/h (0.2 mm/s) for 16 h. To analyze biofilms confocal laser scanning microscopy (CLSM; Zeiss LSM 510 CLSM, Carl Zeiss, Jena, Germany) equipped with a water corrected 63x/NA1.2 objective and filter and detector settings for monitoring GFP fluorescence (excitation at 488 nm using an Ar-laser, emission light selected with a 505–550 nm bandpass filter) was used. Image acquisition was done using the ZEN 2009 software (Carl Zeiss) with z-stack sections of 0.5 µm and three-dimensional reconstruction of z-stacks was performed using the AMIRA software (version 6.0.1, ThermoFisher Scientific). Three runs for each coating variant were carried out.

### 2.5 Controlled implant contamination by bacterial dipping

**FIGURE 1:**
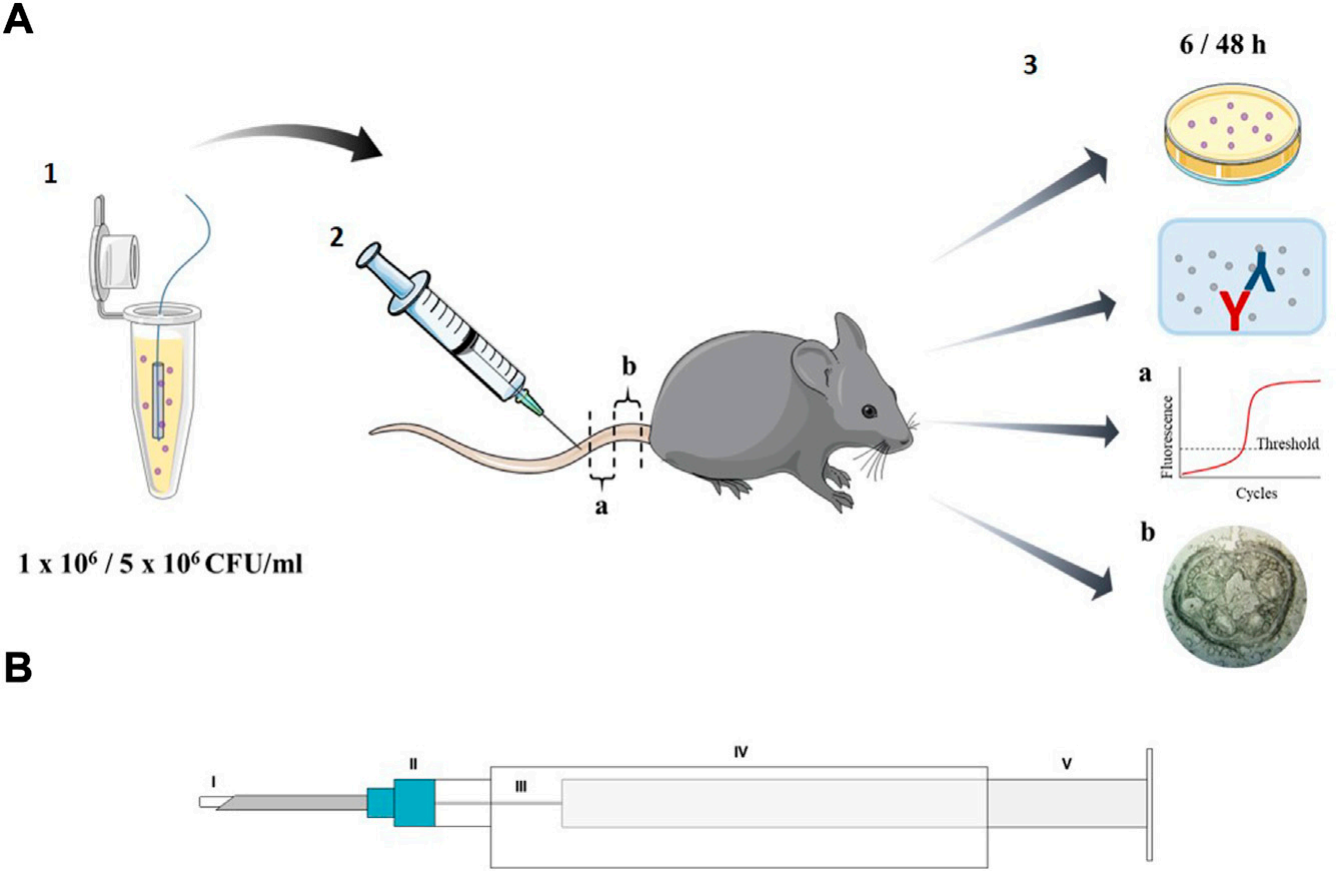
Schematic representation of **(A)** the experimental set-up including (1) contamination of the implant using dipping protocol (2) application of the implants into the tail vein of mice and (3) analysis of the mice local and systemic reaction; (a) part of the tail for qPCR analysis and (b) histology and **(B)** the insertion system containing (I) the implant (II) 21 ^1^/_2_ G needle (III) guide wire, 0.4 mm diameter (IV) 5 ml syringe body (V) 1 ml syringe plunger.

Controlled contamination of the implants for *in vivo* analysis was performed according to a bacterial dipping protocol. For ease of handling, the implants were threaded onto a surgical suture (Prolene 6–0; Ethicon, United States) (Figure 1A). Before dipping, samples were sterilized by short treatment with 70% ethanol. The implants were incubated in two different bacterial dilutions (OD_600_ 0.1 and OD_600_ 0.5) for 30 min while being shaken at 150 rpm. To evaluate the bacterial load on the implants, the bacteria were detached using ultrasonication for 5 min and transferred to agar plates in various dilutions. The colony-forming units (CFU) were counted after overnight incubation. OD_600_ 0.1 was set for a bacterial count of 1 × 10^6^ and OD_600_ 0.5 for 5 × 10^6^ bacterial cells per implant.

### 2.6 Experimental *in vivo* design and implant insertion

Female C57BL/6 mice (8–10-week-old; Janvier Labs, France) were housed in individually ventilated cages with *ad libitum* water and standard chow (R/M-H; Ssniff Spezialdiäten GmbH, Germany) access. One day before implant insertion, metamizol sodium (0.8 mg/500 ml; Ratiopharm, Germany) was administered *via* drinking water to ensure analgesia. The implants were contaminated in a controlled way using the dipping protocol described above. Mice were anesthetized by intraperitoneal injection of ketamine (75 mg/kg; bela-pharm GmbH & Co., KG, Germany) and xylazine (10 mg/kg; Bayer AG, Germany). During the procedure, the mice were kept warm by an infrared lamp. To optimize tail vein puncture, an insertion system was developed to permit easy implantation of the necessary material into the tail vein (Figure 1B). The system consists of a 5 ml syringe body (Discardit; Becton Dickinson, United States), a 1 ml syringe plunger (Omnifix; B. Braun, Germany), an 0.4 mm silver wire (0.4 mm silver wire; Rayher, Germany) attached to the plunger and a 21 ½ G cannula (Microlance™ three; Becton Dickinson, United States). At an appropriate depth of anesthesia, the tail was disinfected and the vein manually compressed. Implants were inserted into the cannula of the insertion system and the proximal third of the lateral tail vein was punctured. The implant was inserted by gently pushing forward the plunger of the syringe with the attached guidance wire. The cannula was removed and the puncture site was compressed to minimize hemorrhage.

The experiment included 12 groups (Table 1). A total of 92 implants (46 coated with rifampicin/minocycline and 46 coated with dalbavancin) were inserted. The implants were either uncontaminated, ligthly contaminated (1 × 10^6^ CFU/implant) or highly contaminated (5 × 10^6^ CFU/implant) and were examined after 6 or 48 h. Mice receiving uncontaminated implants served as controls (n = 10 for experimental groups; n = 3 for control groups).

**TABLE 1:** Experimental groups for tail vein mouse model.

	Implant laytime						
stent graft PLLA coating containing	6 h				48 h		
	Contamination						
		sterile	1 × 10^6^	5 × 10^6^	sterile	1 × 10^6^	5 × 10^6^
rifampicin/minocycline	n	3	10	10	3	10	10
dalbavancin		3	10	10	3	10	10

### 2.7 Systemic bacterial dissemination

Six or 48 h after implant application, the animals were sacrificed in a humane manner for organ analysis. Blood (50 µL) was transferred to agar plates (LB broth with agar, 35 g/L; Sigma-Aldrich, United States) under sterile conditions and CFU counted after overnight incubation. Spleens and kidneys were collected separately in 1 ml phosphate buffered saline (PBS; Thermo Fisher Scientific, Germany) and homogenized using a 70 μm cell strainer (Corning, United States). All samples were washed with PBS and collected in falcon tubes (50 ml). 100 µL of 3 ml spleen homogenates and 5 ml kidney homogenates were transferred to agar plates under sterile conditions and CFU counted after overnight incubation.

### 2.8 Cytokine quantification

To determine the degree of systemic inflammation, cytokines, C-reactive protein (CRP), tumor necrosis factor alpha (TNF-α), interleukin-6 (IL-6) and granulocyte-colony stimulating factor (G-CSF) were measured in pooled blood plasma samples using enzyme-linked immunosorbent assay (ELISA, DuoSet Elisa Kit, R&D Systems, United States) after 6 and 48 h. All ELISAs were performed according to the manufacturer’s instructions. Samples for the CRP ELISA were diluted 1:10^6^ as agreed with the manufacturer. Measurements were carried out in a 96-well plate using a micro-plate reader (FLUOstar Omega, BMG LABTECH GmbH, Germany). Each experimental group was divided into three pools (each with n = 3). The control group consisted of mice which had received sterile, antibiotic-coated implants representing a single pool.

### 2.9 Quantification of gene expression

A defined part of the affected tail section (approximately 0.5 cm, Figure 1A) was separated by macrodissection, snap frozen and stored at −80°C until further processing (no longer than 4 weeks). Total RNA was isolated using the NucleoSpin RNA XS kit (Macherey-Nagel, Germany). In brief, tissue samples were thawed on ice and the tail vein containing the implant was flushed 3 times with 200 µL lysing buffer using a 2 ml syringe with a 21 1/2 G cannula. Further RNA isolation was conducted according to the manufacturer’s protocol. RNA quantity and quality was determined using the Colibri micro-volume spectrometer (Berthold Technologies GmbH & Co.KG, Germany). Reverse transcription of 200 ng RNA into cDNA was performed using RevertAid First Strand cDNA Synthesis Kit (ThermoFisher, Germany) under the following reaction conditions: 25°C for 5 min, followed by 42°C for 60 min and 70°C for 5 min. The total reaction volume was 20 μL, which was diluted by 1:5 after cDNA synthesis. To analyze gene expression, the following TaqMan assays (ThermoFisher, Germany) were used: vascular endothelial growth factor A (*vegfa*; Mm00437306_m1), intercellular adhesion molecule 1 (*icam1*; Mm00516023_m1), von Willebrand factor (*vwf*; Mm00550376_m1), toll-like receptor 2 (*tlr2*, Mm01213946_g1) and glyceraldehyde 3-phosphate dehydrogenase (*gapdh*, Mm99999915_g1) served as endogenous control. RNase-free water was used as contamination control. Gene expression levels were determined *via* quantitative real-time polymerase chain reaction (qPCR) with 5 ng of cDNA in a reaction volume of 20 μL in a 96-well plate (MicroAmp Fast Optical 96-well Reaction Plate, 0.1 ml; ThermoFisher, Germany) using QuantStudio 3 (ThermoFisher, Germany) under the following reaction conditions: 50°C for 2 min followed by 95°C for 10 min, 40 cycles at 95°C for 15 s, and at 60°C for 1 min. Relative gene expression was obtained using the 2^(- ΔΔCt)^ method and determined using Microsoft Excel software (Excel 2016; Microsoft Corporation, United States). Gene expression values were presented as the fold change to the control group (mice receiving uncontaminated implants) normalized to the endogenous reference gene *gapdh*.

### 2.10 Histology

To evaluate the tissue around the implant, a defined part of the affected tail section (approximately 0.5 cm, Figure 1A) was collected in 4% buffered formalin. After 4 weeks of decalcification of the bone material in a decalcifying solution (USEDECALC, Medite Medical GmbH, Germany), the samples were embedded in paraffin. Thin cross sections of 4–6 µm were prepared using a microtome (Hyrax M55 rotary microtome, Carl Zeiss, Germany). Following deparaffinization in xylene and rehydration in a serial dilution of ethanol (100–70%), slides were stained using Trichrome Stain (Masson) Kit (Sigma-Aldrich, United States) according to the manufacturer’s instructions. In this method, the cell nuclei are stained black-blue with hematoxylin, the collagen is stained blue with aniline blue, and the cytoplasm and muscles are stained red with Beibrich scarlet-acid fuchsin. Image acquisition was performed with the Primovert inverted microscope equipped with the AxioCamMRc (Carl Zeiss, Oberkochen, Germany).

### 2.11 Statistics

Statistical analysis was performed using GraphPad Prism 9.1 (GraphPad Software, United States). Values are expressed as mean with SD. Normal distribution was tested using the Shapiro-Wilk test. Normally distributed data were compared using one-way ANOVA and for non-normally distributed data Kruskal–Wallis test was used. For all statistical analyses, *p* values < 0.05 were considered significant (*p < 0.05; **p < 0.02; ***p < 0.001). Mice that received sterile implants served as the control group and were used as the reference.

## 3 Results

### 3.1 Both the rifampicin/minocycline combination and dalbavancin exhibit sufficient integration into the surface coating

**FIGURE 2:**
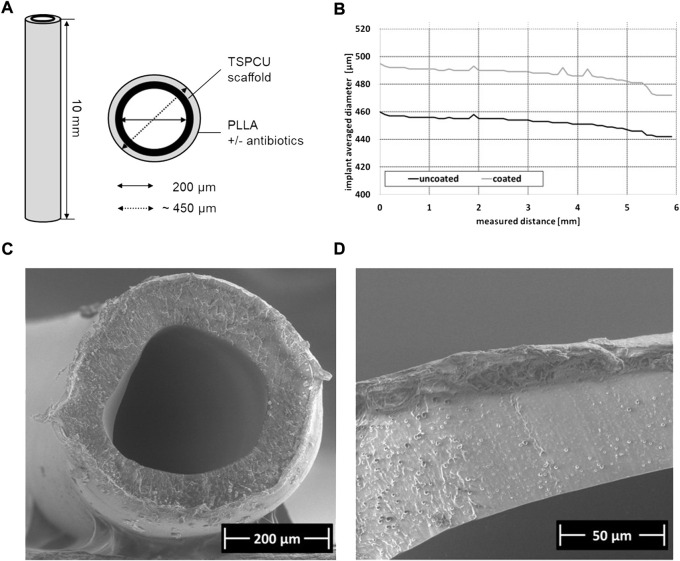
Characterization of implants samples **(A)** Schematic representation of implant design, **(B)** exemplary measurement of the implant average diameter by biaxial laser scanner, **(C)** Scanning electron micoscropy (SEM) image of implant cross section with PLLA coating containing rifampicin/minocycline; magnification ×200, **(D)** SEM detailed image of the PLLA coating containing rifampicin/minocycline; magnification ×800.

The tubes used for implantation had an original luminal diameter of approximately 200 μm, an original outer diameter of 450 μm, and were cut to a length of approximately 10 mm (Figure 2A). After the coating process, the biaxial laser scanner revealed a uniform coating thickness of about 150 μm, which slightly decreased the greater the measuring distance (Figure 2B). Representative SEM analysis of a rifampicin/minocycline-coating confirmed smooth and uniform coating of the implant stent grafts (Figure 2C, D).

### 3.2 Both rifampicin/minocycline and dalbavancin inhibit *Staphylococcus aureus* FR20 biofilm formation *in vitro*

**FIGURE 3:**
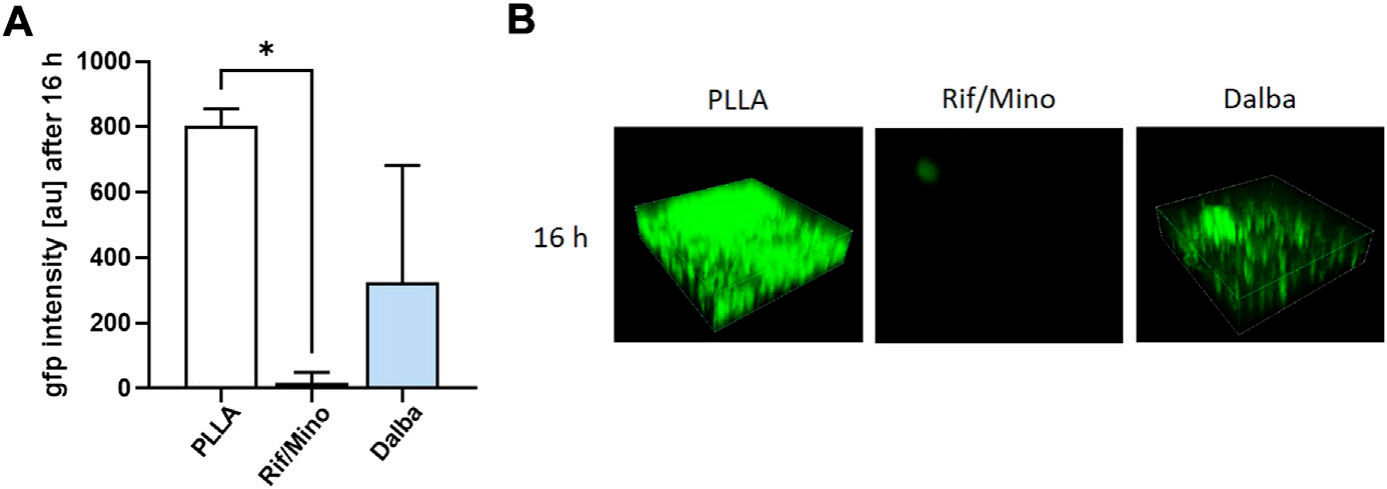
Rifampicin/minocycline and dalbavancin inhibit the biofilm formation of *Staphylococcus aureus* FR20. The effectiveness of rifampicin/minocycline and dalbavancin against *Staphylococcus aureus* FR20 biofilm formation was analyzed in a flow system. Inhibition of biofilm formation was monitored by **(A)** measuring green fluorescent protein (GFP) fluorescence intensity and **(B)** confocal laser scanning micoscropy (CLSM) of randomly selected areas (spanning 100 μm × 100 µm) after 16 h *p* values <0.05 were considered significant. *p < 0.05. Rif/Mino: rifampicin/minocycline; Dalba: dalbavancin; au: arbitrary unit.

The *in vitro* effectiveness of rifampicin/minocycline and dalbavancin to inhibit the ability of *S. aureus* FR20 to form biofilms was analyzed in a flow system using PLLA-coated glass slides loaded with the different active agents. For this purpose, *S. aureus* FR20 was modified with GFP, which allowed determination of fluorescence intensity and visualization in CLSM. After 16 h, GFP fluorescence intensity was significantly reduced in samples coated with rifampicin/minocycline. A reduction of approximately 60% was observed in the dalbavancin samples (Figure 3A). These results were confirmed by representative CLSM images, which visualizes the reduced biofilm formation on the samples containing rifampicin/minocycline or dalbavancin by decreased fluorescence intensity (Figure 3B).

### 3.3 Antibiotic coatings reduce the bacteria-induced systemic immune response in the host

**FIGURE 4:**
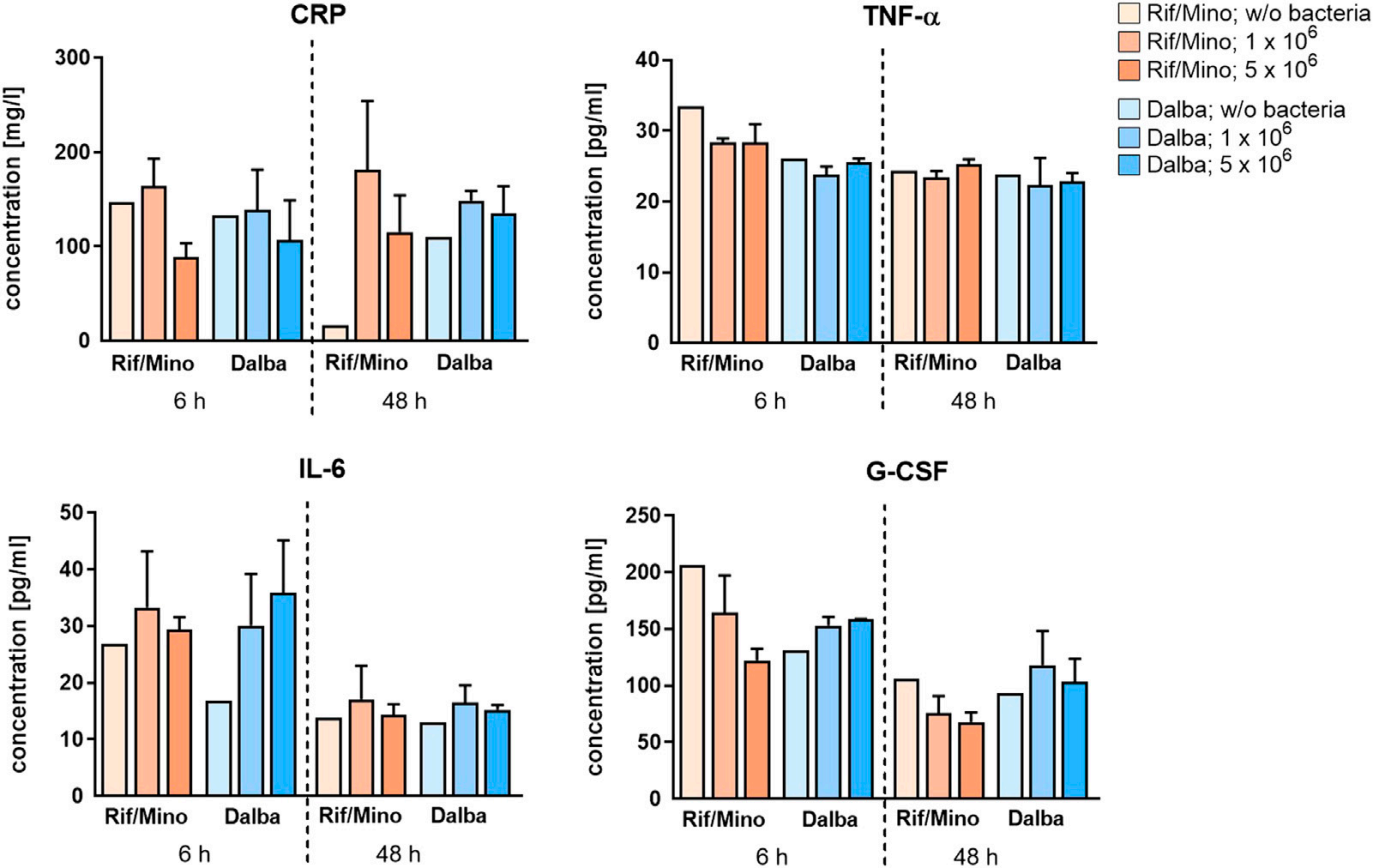
Antibiotic coatings reduce the bacteria-induced systemic immune response in the host. The concentration of cytokines (CRP, TNF-α, IL-6 and G-CSF) in pooled blood plasma was quantified using ELISA after 6 and 48 h. For CRP detection, samples were diluted 1:10^6^ in recommended reaction buffer. Data are represented as mean + SD. Rif/Mino: rifampicine/minocycline; Dalba: dalbavancin.

*In vivo*, bacterial clearance based on the dissemination of bacteria in the blood, spleen, and kidneys of mice was studied using the pour-plate method to determine CFU. Almost no CFU were detected in the organic materials for any of the time points in the rifampicin/minocycline group with the exception of 10 CFU in the spleen of one mouse in the low infected group after 6 h as well as 10 CFU in the spleen of one mouse in the low contamination group after 48 h. In the dalbavancin group, CFU were also only detected in exceptional cases which occurred independently of the severity or duration of the infection (data not shown). Cytokine release (CRP, TNF-α, IL-6, G-CSF) in blood plasma was also analyzed in order to evaluate the host systemic response towards the contaminated implant (Figure 4). No consistent time-related changes in CRP were observed in either the rifampicin/minocycline or dalbavancin groups. In the rifampicin/minocycline group, the high contamination subgroups showed a decrease of approximately 45.9% at the early time point and of 36.3% at the later time point compared with the low contamination subgroups. Another striking result obtained for the rifampicin/minocycline coating was the 8-fold decrease in CRP in the sterile group at 48 h compared with the sterile group at 6 h. A slight time-dependent decrease in TNF-α was revealed in both coating variants. IL-6 release showed a remarkable decrease with highest levels (35.9 pg/ml) in the high contamination dalbavancin-coated group at 6 h to levels around 15 pg/ml on average for all other groups at 48 h. This effect was also observed for G-CSF, which declined by approximately 53.9% (low contamination)/44.6% (high contamination) in the rifampicin/minocycline groups and 22.9% (low contamination)/34.7% (high contamination) in the dalbavancin groups after 48 h compared to the respective 6 h analog. In general, there was no difference between the contamination levels with regard to their effect on the release of any of the cytokines analyzed.

### 3.4 Antibiotic activity decreases gene expression associated with local inflammatory reaction towards the implant

For a molecular evaluation of the local inflammatory response to the implant, RNA was isolated and qPCR performed. The gene expression of *vegfa*, *icam1*, *tnf-α*, *tlr2* and *vwf* was analyzed. *Vegfa* displayed a time-dependent decrease of about 69.1% (low contamination) and 52.9% (high contamination) in the rifampicin/minocycline groups and about 35.9% (high contamination) in the dalbavancin groups. The contamination levels did not cause significant differences in expression levels for this gene. *Icam1* was reduced in all groups after 48 h. Gene expression was increased at the early time point in the dalbavancin groups compared with the rifampicin/minocycline groups, but then significantly decreased approximately 10-fold after 48 h. For *tnf-α*, a significant decrease was observed in the low contamination rifampicin/minocycline group between 6 and 48 h. The low contamination dalbavancin group showed the highest expression levels of tnf-*α* at 48 h and significantly increased the levels 13-fold compared with the low contamination rifampicin/minocycline group at 48 h. *Vwf* showed a time-dependent decrease of 62.6% (rifampicin/minocycline, low contamination) up to 80.1% (dalbavancin, high contamination) across all groups, except of the low contamination dalbavancin group, which revealed increased expression. *Tlr2* decreased over time for the rifampicin/minocycline coating, with a significant 10-fold reduction observed in the low contamination group. No significant changes in the expression of this gene were detected in the dalbavancin group. Antibiotic activity decreases gene expression associated with local inflammatory tissue response towards the contaminated implants.

**FIGURE 5:**
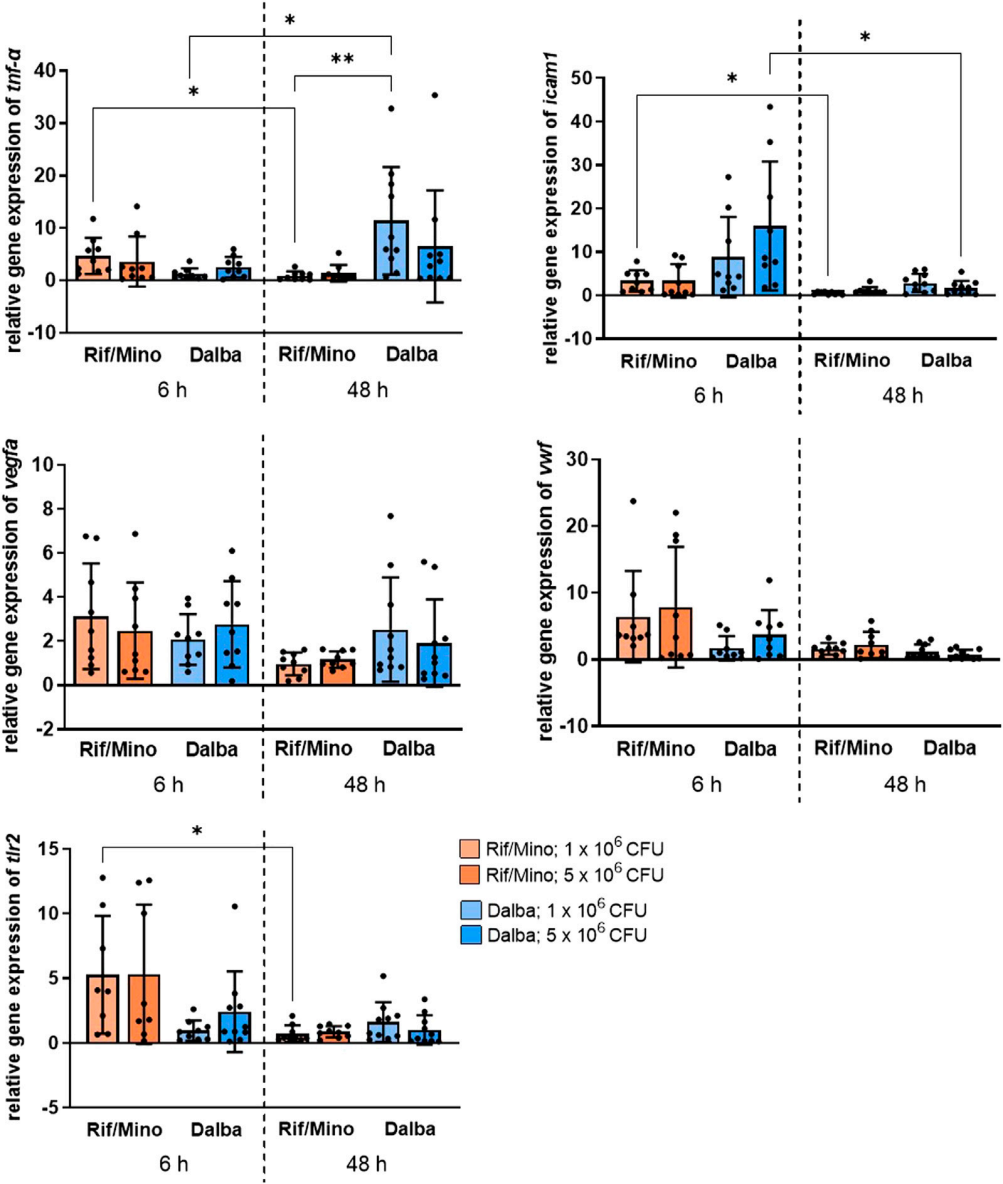
Antibiotic activity decreases the local inflammatory tissue response towards the contaminated implants. Relative gene expression levels of *vegfa*, *icam1*, *tnf-α*, *vwf* and *tlr2* were determined by qPCR. Data are represented in scattered dot plots with mean + SD. *p* values <0.05 were considered significant. *p < 0.05; **p < 0.02.

For the purpose of implant localization and to assess the degree of infection, histological trichrome staining was carried out on the part of the tail where the implant was inserted. The implants could be detected at both time points (6, 48 h) as a definite roundish demarcation from the surrounding tissue. Further examination did not reveal any major histological differences between the rifampicin/minocycline and the dalbavancin groups (Figure 5). Unlike in the study by Moerke et al. (Moerke et al., 2021), in which sterile and contaminated implants were used without antibiotic coating, considerable differences were observed between the groups in terms of the formation of inflammatory aggregates and the disintegration of surrounding tissue (Figure 5).

## 4 Discussion

The aim of the present study was to evaluate in a minimally invasive mouse model the efficacy of a novel antibiotic coating based on dalbavancin as a long shelf-life single agent compared with a well-known local drug depot of rifampicin/minocycline. To adequately assess the biological mode of action of implant modifications, we simulated conditions of bacterial vascular implant infection. Small polymeric tubes were subjected to controlled contamination with *Staphylococcus* (*S.*) *aureus* FR20 before insertion into the tail vein of mice. For contamination purposes, we developed a bacterial dipping protocol to ensure the test samples received a uniform and purely bacterial load. The tubes were made of TSCPU, a polycarbonate-based polyurethane with a 5% silicone content. TSCPU is a biocompatible material with high pressure resistance, high tensile strength and high chemical resistance (Zdrahala and Zdrahala, 1999). The silicone content provides increased elongation, elasticity and a low coefficient of friction (Bazan et al., 2019). The tube coating was based on poly-L-lactid (PLLA), into which the antibiotics rifampicin/minocycline or dalbavancin were incorporated. PLLA is a hydrophobic polymer known for its good biocompatibility and processability (Tsuji et al., 2006; Vroman and Tighzert, 2009). Due to its biodegradability, it is suitable for a variety of drug delivery applications and a useful tool in achieving better anti-microbial performance (Gollwitzer et al., 2003; Santoro et al., 2016). Qureshi et al. demonstrated the anti-microbial effect of PLLA when used to release small silver nanoparticles (SNPs) (Qureshi et al., 2014), and Kälicke et al. investigated the efficacy of PLLA coatings with an antiseptic or antibiotic content, including a combination of rifampicin and fusidic acid, on titanium osteosynthesis implants (Kälicke et al., 2006). While it has already been shown that coating catheters with the antibiotic combination rifampicin/minocycline is successful against bloodstream infections (Reitzel et al., 2020) and the colonialization of catheters with staphylococci (Raad et al., 1995; Raad et al., 1996), dalbavancin has never been used in a drug delivery system (DDS) so far. Dalbavancin belongs to the group of glycopeptide antibiotics that inhibit the cell wall synthesis of Gram-positive bacteria and is indicated for acute bacterial skin and soft tissue infections (Barberán et al., 2021). It has demonstrated a potent anti-microbial effect both in infection models (Jabés et al., 2004) and various real-life bacterial infections (Bouza et al., 2018; Almangour et al., 2019). Moreover, compared to vancomycin, dalbavancin has been shown to be more protective against *S. aureus* colonization of devices *in vivo* (Darouiche and Mansouri, 2005) and to reduce biofilms of methicillin-resistant *S. aureus* (MRSA), methicillin-resistant *S. epidermidis* (MRSE) and vancomycin-resistant enterococci (VRE) (Streit et al., 2004; Knafl et al., 2017). This effectiveness against difficult-to-treat bacteria together with its relatively long half-life of two weeks makes it a promising candidate for implant coatings.

In the present study, small polymer tubes were coated using a system which incorporates rifampicin/minocycline and dalbavancin into a PLLA matrix. The success of the coating process was confirmed by measuring the implant diameter using a biaxial laser scanner and scanning electron microscopy (SEM) (Figure 2). *In vitro* testing of both the rifampicin/minocycline and dalbavancin coatings demonstrated effectiveness against biofilm formation of *S. aureus* FR20 (Figure 3A, B). *In vivo*, we were able to confirm the bactericidal effect of the implant coatings. Hardly any bacteria were detected in the blood samples, spleens or kidneys of the animals from the rifampicin/minocycline group. In the dalbavancin group, the same trend was observed (data not shown). However, in some mice, bacteria colonies could be detected in the blood, spleen, and kidney regardless of the level of contamination. This is possibly a result of the processing of the dalbavancin coating. Uniformly coating the dalbavancin samples was challenging as dalbavancin precipitates at a temperature below 25°C, but the spray coating system uses pressurized cold air to apply the substances to the tubes. The system was adjusted for this reason, but the possibility of errors cannot be excluded. To us, it seems more plausible that this sporadic occurrence of colonies in homogenized organs was due to contamination during the explantation process, since the inflammation levels were not conspicuous in the animals concerned. Moreover, the antibiotic activity of the drugs used has already been demonstrated: rifampicin/minocycline is known to reduce bloodstream infections (Reitzel et al., 2020) and has been shown to be highly effective against staphylococcal colonization of catheters (Raad et al., 1995; Raad et al., 1996) while dalbavancin has been shown to be effective against MRSA and MRSE in growing biofilms *in vitro* (Streit et al., 2004; Knafl et al., 2017). However, it has never yet been applied in coating systems.

Cytokine levels in the plasma of the mice were analyzed to characterize the host systemic immune response (Figure 4). The acute-phase protein CRP, which is increased in infectious and some cardiovascular diseases, is a biomarker of inflammation (Du Clos and Mold, 2004; Sproston and Ashworth, 2018). The baseline concentration in adult wild-type C57BL/6 mice was reported by Simons et al. to be 5–9 mg/L (Simons et al., 2014). The approximately 10-fold elevated concentrations of CRP found in this study thus indicate only a small degree of inflammatory activation as CRP is known to increase more than 1000-fold in the presence of infection (Teupser et al., 2011). This is in line with plasma levels of tumor necrosis factor α (TNF-α), which were also within the physiological range of 10–250 pg/ml (Abram et al., 2000; Stenina et al., 2012). Plasma levels of interleukin 6 (IL-6) and granulocyte-colony stimulating factor (G-CSF) showed a time-dependent reduction. TNF-α and G-CSF are inflammatory markers that can be induced by bacterial colonization, among other factors. In the acute phase of an inflammatory process, TNF-α and G-CSF increase, with G-CSF decreasing again when the neutrophil host response is attenuated (Moser et al., 2017). Mice lacking the ability to produce and release G-CSF and IL-6 have been found to be more susceptible to *Candida* infections, for example (Basu et al., 2008). IL-6, a pro-inflammatory cytokine, is also secreted during the initial stages of inflammation by various cells such as inflammatory cells, keratinocytes, fibroblasts, and endothelial cells. This process, in turn, induces the release of a number of acute phase proteins, including CRP (Zhang and An, 2007; Tanaka and Kishimoto, 2014). Our results suggest an initial, although barely present, systemic inflammatory response that almost completely subsides with time. The lack of difference between the immune reactions to the different levels of contamination could be due to the sheer effectiveness of the antibiotics, allowing them to perform comparably in combating both levels of bacterial load.

**FIGURE 6:**
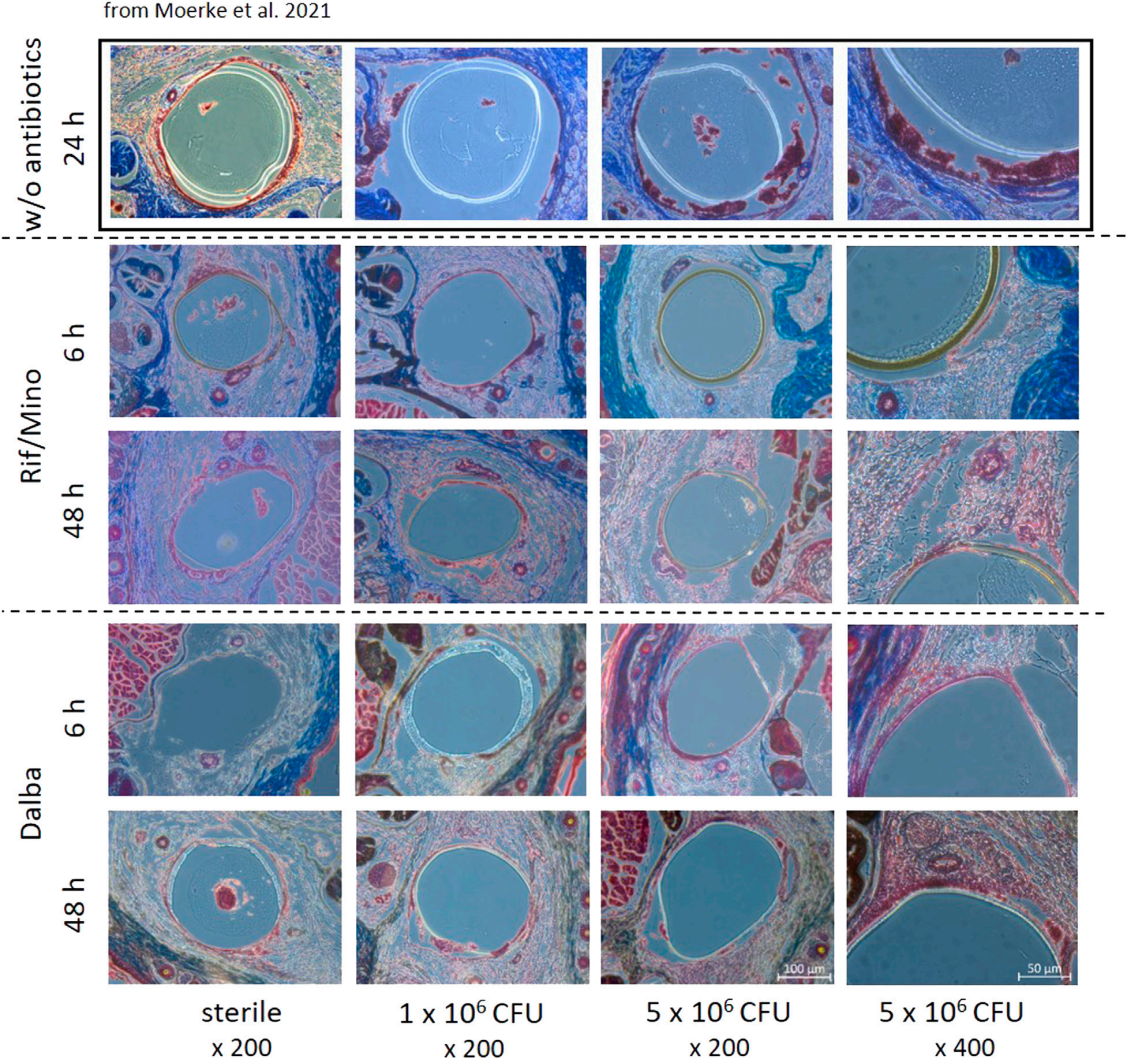
Histological trichrome staining of tail cross sections was performed after 6 and 48 h. Representative images of mice tails that received implants without antibiotic coating (excerpted from Moerke et al. (Moerke et al., 2021)), coated with rifampicin/minocycline, and dalbavancin are shown; bars 100 µm or 50 μm; Magnification, × 200 or x 400. Different color expression is due to performing the staining on different days. Rif/Mino: rifampicine/minocycline; Dalba: dalbavancin.

In line with plasma cytokine levels, a perceptible decrease in the expression of genes associated with endothelial activation and local inflammation (*vegfa*, *icam1*, *tnf-α*, *tlr2*, *vwf)* was observed in the tissues surrounding the implant in both the rifampicin/minocycline and dalbavancin groups (Figure 6). A notable difference between the two antibiotic coatings was found for two genes: *icam1* expression was about five times higher in the dalbavancin group compared with the rifampicin/minocycline group after 6 h, and *tnf-α* exhibited an up to a 10-fold increase after 48 h. This might suggest that dalbavancin coating induces increased endothelial activation. A similar phenomenon has in fact been observed for dalbavancin and *vegfa* by Simonetti et al. (Simonetti et al., 2020). The decreasing expression of *vwf* and *tlr2* over time indicates the reduction of the bacteria-induced inflammatory response. This is confirmed by the correlation of TLR2 in staphylococcal peptidoglycan and lipoteichoic acid (LTA) recognition (Fournier and Philpott, 2005; Brandt et al., 2018). Similar findings have been reported for vWF, as showed that attachment of *S. aureus* to blood vessels occurs under the influence of vWF (Claes et al., 2014; Claes et al., 2017). One reason for the observed large standard deviations and lack of significance could be due to poor RNA integrity. However, the decreasing inflammation observed in the experimental groups was also reflected in the histology of mice tail samples (Figure 5). Trichrome staining showed an intact vascular endothelium at both time points examined, with no evidence of a local inflammatory reaction. In contrast, without antibiotics a marked inflammatory reaction was observed (Figure 5) (Moerke et al., 2021).

In addition to achieving promising results regarding the effectiveness of a novel dalbavancin coating *versus* rifampicin/minocycline, we were able to validate an *in vivo* model for bacteria-induced implant infection. So far, bacterial infections of implants have often been induced for study either by systemic administration of bacteria (Wang et al., 2017), by concurrent inoculation at the implantation site (BARTH et al., 1989; Roehrborn et al., 1995), or by *ex post* inoculation days after implant placement and the healing of the incision (Roehrborn et al., 1995). In the latter case, a second surgical intervention is required. In addition, systemic (i.e., intravenous) administration of bacteria is a delicate issue due to the risk of mice developing bacteremia or even sepsis associated with high disease burden. In the *in vivo* model presented here, the local bacterial contamination minimizes the disease burden for the animals. An additional advantage of the tail vein method is the fact that material can be inserted in a minimally invasive way without the need for surgical intervention or additional fixation. Nevertheless, this study also has limitations that should be mentioned. Although we have been able to show that bacterial spread originating from the implant can be prevented, we do not know what would happen in infections caused by bloodstream bacteria. Thus, it can be concluded that the model’s applicability is limited to investigations of early implant infections. Further research to expand the range of applications, such as the mode of infection or improvement of coating systems, needs to be pursued.

## 5 Conclusion

In the present study, we successfully created minimally invasive implantable antibiotic-loaded microtubes and thus were able to implement an innovative drug-releasing coating system. We demonstrated the anti-infective efficacy of a novel dalbavancin coating, which is similar in potency to the combination rifampicin/minocycline coating. Both coatings were shown to have excellent activity against hematogenous spread of bacteria and to prevent local tissue reaction to the contaminated implant. To our knowledge, the placement of an antibiotic-coated implant has not been combined with direct blood contact and local bacterial challenge through controlled implant contamination so far.

## Data Availability

The original contributions presented in the study are included in the article/supplementary material, further inquiries can be directed to the corresponding author.
